# A Three Step Network Based Approach (TSNBA) to Finding Disease Molecular Signature and Key Regulators: A Case Study of IL-1 and TNF-Alpha Stimulated Inflammation

**DOI:** 10.1371/journal.pone.0094360

**Published:** 2014-04-18

**Authors:** Jihong Yang, Zheng Li, Xiaohui Fan, Yiyu Cheng

**Affiliations:** 1 Pharmaceutical Informatics Institute, College of Pharmaceutical Sciences, Zhejiang University, Hangzhou, China; 2 State Key Laboratory of Modern Chinese Medicine, Tianjin University of Traditional Chinese Medicine, Tianjin, China; UT MD Anderson Cancer Center, United States of America

## Abstract

A disease molecular signature is a set of biomolecular features that are prognostic of clinical phenotypes and indicative of underlying pathology. It is of great importance to develop computational approaches for finding more relevant molecular signatures. Based upon the hypothesis that various components in a molecular signature are more likely to share similar patterns, we introduced a novel three step network based approach (TSNBA) to identify the molecular signature and key pathological regulators. Protein-protein interaction (PPI) network and ranking algorithm were integrated in the first step to find pathology related proteins with high accuracy. It was followed by the second step to further screen with co-expression patterns for better pathology enrichment. Context likelihood of relatedness (CLR) algorithm was used in the third step to infer gene regulatory networks and identify key transcription regulators. We applied this approach to study IL-1 (interleukin-1) and TNF-alpha (tumor necrosis factor-alpha) stimulated inflammation. TSNBA identified inflammatory signature with high accuracy and outperformed 5 competing methods namely fold change, degree, interconnectivity, neighborhood score and network propagation based approaches. The best molecular signature, with 80% (40/50) confirmed inflammatory genes, was used to predict inflammation related genes. As a result, 8 out of 10 predicted inflammation genes that were not included in the benchmark Entrez Gene database were validated by literature evidence. Furthermore, 23 of the 32 predicted inflammation regulators were validated by literature evidence. The rest 9 were also validated with TF (transcription factor) binding site analysis. In conclusion, we developed an efficient strategy for disease molecular signature finding and key pathological regulator identification.

## Introduction

Molecular signature is defined as a set of biomolecular features that can be used as markers for a particular phenotype and underlying condition-related biological mechanisms. They can be a set of genes, proteins, metabolites, genetic variants and microRNAs. Molecular signatures have been derived and applied for various purposes [1], [2] including disease diagnosis and risk assessment [3]–[7], prediction of physiological toxicity [8], [9] and response to therapeutic drugs [10], [11]. In addition, molecular signatures are also indicative of underlying molecular pathology and have been used for investigating disease progression [12], [13] and discovering the underlying mechanisms [14], [15].

Molecular signature can be obtained via a variety of approaches. Dimension reduction techniques [16], [17], differential expression analysis [18], and prioritization approaches [19], [20] are commonly used for this purpose. However, signature components obtained from principal component analysis (PCA) and partial least squares (PLS) are often difficult for interpretation. In addition, reproducibility and accuracy are still two challenges for current methods. “Omics” technologies have produced a lot of high throughput data, which provides tremendously rich information to discover molecular signature for better understanding diseases. In addition, diverse types of data can be integrated in network based approaches, which advantageously incorporate complex interactions and rich disease information. Methods integrating multiple data sets, multiple data types with network-based approaches have been shown to find accurate and robust molecular signatures [1].

Another major challenge still exists regarding the lack of robustness for the algorithms with overly optimistic result for certain data sets and poor performance on other data sets. Different stimulations may lead to similar clinical phenotype by perturbing very different underlying molecular mechanisms. Therefore, it is important to improve current discovery process for identifying perturbation responsive signatures. Moreover, considering the experimental validation of a signature, it is more important to reduce hundreds of signature proteins/genes to a refined and manageable number of key regulators. Therefore, it is useful to develop an approach for accurate molecular signature and pathological regulators discovery at the same time.

**Figure 1:**
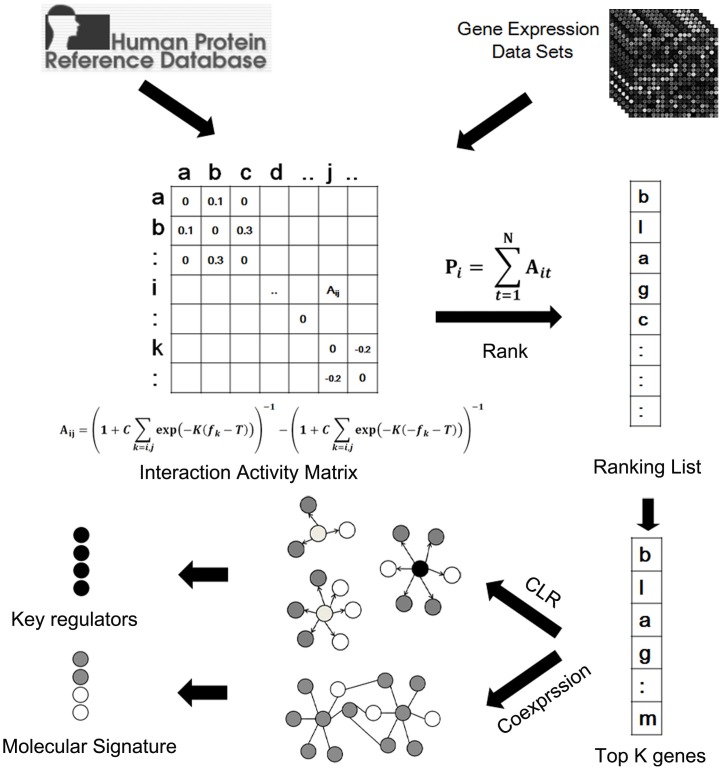
Framework of TSNBA. PPI network and gene expression data are integrated in the interaction activity matrix to rank genes for their relevancy to the perturbation. The top K ranking genes are further filtered with co-expression network for better pathology enrichment. Context likelihood of relatedness (CLR) algorithm is used in the third step to infer gene regulatory networks and identify key transcription regulators. Node in gray represents for known pathology related genes, white represents for predicted ones, and black represents for predicted key regulators.

It is well recognized that interacted genes or proteins are likely involved in the same or similar biochemical process [21], [22]. Therefore, similar expression patterns are more likely to be shared by components involved in the same molecular signature for a specific pathological process. Based on this understanding, a new approach was developed for finding disease molecular signature and key regulators by integrating PPI network, gene co-expression network and context likelihood of relatedness (CLR) algorithm as shown in Figure 1. In the first step, TSNBA (three step network based approach) uniquely combined gene expression data with PPI network to find pathology related proteins through a novel ranking algorithm incorporating perturbation responsive gene expression data. In the second step, the top ranking genes were further screened with co-expression network for a more enriched signature finding. Finally, CLR algorithm was used for inferring gene regulatory networks, followed by identification of key regulators based upon three screening criteria. The performance of TSNBA was tested on IL-1 (interleukin-1) and TNF-alpha (tumor necrosis factor-alpha) stimulated inflammation. As a result, TSNBA outperformed 5 competing methods namely fold change, degree, interconnectivity, neighborhood score and network propagation based approaches. The predicted pathological regulators were validated with literature evidence and provided potential new insights into the underlying molecular mechanisms of inflammation.

## Materials and Methods

### Data Preparation

#### PPI data

The PPI data was derived from HPRD [23] (Release 9) with self-interactions removed.

#### TNF-alpha and IL-1 stimulated inflammation data

The former, GSE2639 [24], contains the gene expression profiles of 4 TNF-stimulated samples and 4 normal samples. Stimulated samples were treated with 2 ng/ml TNF for 5 hours, and normal samples were left-untreated. The latter, GSE973 [25], contains 4 IL-1 stimulated samples and 1 normal sample. Stimulated samples were treated with 100 U/ml human IL-1 for 0, 0.5, 1, 2.5 and 6 hours, and the normal sample was untreated. Two groups of data sets were both obtained by using human U133A GeneChips (Affymetrix, Santa Clara, CA) from human umbilical vein endothelial cells (HUVEC). Raw data was stored in ArrayTrack 3.5.0 [26]. MAS 5 [27], which has been suggested to be the best normalization procedure to reconstruct cellular network [28], was used for normalization. Expression data was summarized to the gene level by averaging all probes mapped to the same gene. Only the genes included in the PPI network were selected for further analysis.

#### PPI data Benchmark human inflammatory genes

The data was collected from Entrez Gene database [29]. We queried “((“inflammatory” OR “inflammation”) AND “[Homo sapiens (human)]”)” and found 2210 related genes as of 5th September 2013. Only 1462 genes were involved in PPI network and those were used for further analysis.

#### Human TF (transcription factor) data

Human transcription factor data was derived from AnimalTFDB [30], and only TFs involved in PPI network were considered for further analysis.

### The First Step: Gene ranking with PPI interaction network

#### Generation of an interaction activity matrix

An adjacency matrix Adj was constructed for PPI network. The Adj(i,j) = 1 when node i and j interact with each other and Adj(i,j) = 0 otherwise. The activity of each interaction was computed by a weighting function [31], [32]:
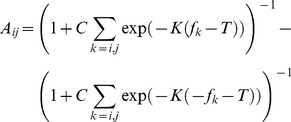
(1)Here, *f_k_* is the log2-fold change value of the gene *k*. The shape of the multivariate logistic distribution is controlled by parameters *C* and *K* (*C* = 1 and *K* = 5 by default), and the shifting parameter *T* (0.5 by default) is added to produce zero when *f_i_* and *f_j_* are both zeros. The weighting function includes two multivariate logistic functions, with the first term capturing co-activation of genes and the second term capturing co-suppression. These activities of interactions replace “1” in Adj and generate an interaction activity matrix.

#### Gene ranking

The influence of each node (*P_i_*) is the sum of the influence it receives from each interacted nodes.
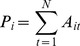
(2)


Where *N* is the number of nodes in the interaction activity matrix, and *A_it_* is the interaction activity of node *t* with node *i*. The final ranked list was obtained according to descending order of *P_i_*.

### The Second Step: Filtering with co-expression network for better enrichment

Gene co-expression has been widely used for finding co-regulated genes [33]–[36] and co-regulatory relationships [37]–[41]. Here, co-expression analysis was applied to the top ranking genes obtained from the first step to further screen for better pathology enrichment. Pearson correlation coefficient *p* was computed for each pair of genes:
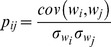
(3)with *w_i_* as the vector containing expression values of gene *i* under all conditions. Correlation coefficients below a certain threshold will be filtered out, and eligible connections are included in the final co-expression network and deemed as the disease molecular signature.

### The Third Step: Searching for key regulators

#### Ranking putative interactions by CLR

A lot of approaches have been developed to identify regulatory networks, such as CLR [42], Bayesian network [43] and ARACNe [44]. As an extension of relevance networks approach [45], [46], CLR detects regulatory interactions via important mutual information (MI). An adaptive background correction step is further applied to eliminate false correlations and indirect influences. The statistical likelihood of MI value for each gene is computed within its network context, and the MI value for each TF-target pair is compared to the context likelihood of both the TF and the target gene, followed by z-score normalization.
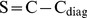
(4)

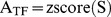
(5)

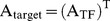
(6)

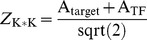
(7)

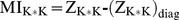
(8)Where C is the correlation coefficient matrix for top K genes of ranking list. C_diag_ is the diagonal matrix of C. MI_K*M_ is derived from MI_K*K_ for M genes coding for transcription factors in top K genes. Finally, putative regulatory interactions are then ranked by decreasing z-score.

#### Identifying key transcription factors of inflammation

Key regulators should be closely associated with the pathology and play pivotal roles in the regulatory network. In the present study, three screening criteria were used for searching key inflammatory regulators. All interactions with z-score less than 3 are filtered out, the number of targets from Entrez Gene inflammation signature must be greater than 3, and the percentage of inflammatory gens in all targets should be more than 60%.

### Competing approaches

#### Fold change based approach

Gene expression change quantifies the response to a pathological condition for a gene. Therefore, differentially expressed genes using a fold change cutoff has been used very often as the signature under the condition. In this study, absolute fold change cutoff was set to be 1.2, 1.5, 2.0, 3.0 and 4.0, eligible genes were collected in corresponding sets, namely “FC_1.2”, “FC_1.5”, “FC_2”, “FC_3”, and “FC_4”. Moreover, top 50 and 300 genes with biggest absolute fold change were collected and corresponding enrichment ratios were calculated.

#### Degree based approach

Degree is an important topological parameter, e.g. hub genes are the genes with highest degrees. Deletion of these genes has been shown leading to more severe phenotypic outcomes than other genes [47]. Although the importance of hub genes are still in debate, they have been used widely as a measure of biological importance. Moreover, our first step ranks genes via the weight calculated by equation (2), which may inadvertently capture the degree information. Thus, it is necessary to compare with degree based approach. According to the PPI network, genes were ranked by decreasing degrees. In the present study, degree cutoff was set to be 50, 100, 150, 200, 250 and 300 and corresponding enrichment ratios were calculated.

#### Neighborhood Scoring

Neighborhood scoring is a local measure for prioritizing candidates based on the expression of the gene itself and its direct neighbors in the network [48], we implemented the adapted method as described in Dorothea Emig's work [49]. Genes were ranked by their scores, which were calculated as follows:
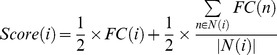
(9)Fold change (*FC*) of gene *i* and average fold change of its neighbors *N* equally contribute to the score, where *N*(*i*) includes all neighboring genes of *i*. To note, score 0 is assigned to genes that are neither differentially expressed or have any differently expressed genes in the direct neighborhood.

#### Interconnectivity

Interconnectivity is also a local measurement for prioritizing candidates, which is based on genes' overall connectivity to differentially expressed genes [50]. An adapted method [49] is used in the present study. First, interconnectivity scores for interactors of differentially expressed genes are calculated based on their direct interactions and their shared neighborhood as follows:
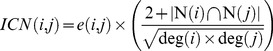
(10)
*e*(*i,j*) describes whether an edge exists between gene *i* and *j*, 1 represents for edge exists and 0 otherwise. Both direct interaction and shared neighborhood N are taken into account, which are then normalized by the overall degrees of the two genes.

Then, final score of each gene is based on the interconnectivity to all differentially expressed genes:
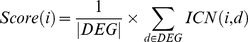
(11)where *DEG* is the set of all differentially expressed genes and *d* represents one differentially expressed gene.

#### Network Propagation

Different from interconnectivity and neighborhood score, network propagation is a global method, which takes the complete network topology into account for prioritizing candidates [49], [51]. First, differentially expressed genes in the network are assigned to a score of 1, while the remaining 0. These scores represent the prior information on genes for disease and are regarded as the starting propagating flow. Then, the flow is further smoothed over the network in each iteration until a steady state is reached. Finally, each gene receives its final score according to the final flow and is ranked in the whole gene list. In each iteration, the flow for the genes is updated as follows:
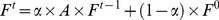
(12)
*F^t^* is a vector containing the flow for each gene at time point *t*. α is diffusion parameter. *A* is the adjacency matrix of the network, where each entry is normalized by the degrees of the source gene and target gene. The normalization compensates for the fact that high degree genes have a higher chance of picking up flow by chance and are thus ranking higher in the prioritization. *F^0^* represents the starting propagating flow. The steady state is reached when the L_1_ norm of the difference between *F^t^* and *F^t-1^* is below 10^−6^.

### Enrichment ratio and statistical test for inflammation

#### Enrichment ratio and statistical test

Enrichment ratio in the present work was defined as the percentage of inflammatory genes in top **K** genes of ranking list overlapping with the benchmark Entrez Gene inflammation set. **K** started from 50 with an increment of 50 at each step. For each ratio, a hypergeometric test was used to evaluate the enrichment of inflammatory genes in each selected list, and p-value was obtained.

#### Enrichment ratio and statistical test for signature genes by TSNBA

Top **K** genes were chosen from the ranked list for screening signature proteins for better pathology enrichment. Different thresholds of correlation coefficient were tested, ranging from 0.6 to 0.945 with an increment of 0.005. For each threshold, genes involved in the co-regulatory relationships were selected to calculate enrichment ratio, and hypergeometric test was used for enrichment analysis.

## Results

### TSNBA identified better inflammation enriched signature

The final PPI network used in this study consisted of 7633 genes (nodes) and 30995 interactions. 1469 human TFs derived from AnimalTFDB database and 1462 inflammatory genes extracted from Entrez Gene database were included in the network. The background ratio for inflammatory gene was 19.2% (1462/7633). Endothelial cells play critical roles during the inflammatory response [25], and TNF-alpha [52] and IL-1 [53] are well known important mediators of the process. Therefore, gene expression data collected from TNF-alpha and IL-1 stimulated HUVEC were used in the present study. For IL-1 stimulated inflammation, HUVEC were treated with IL-1 for 0, 0.5, 1, 2.5 and 6 hours, 4 sets of data were constructed to calculate fold change of genes, namely “IL1_0.5h”, “IL1_1h”, “IL1_2.5h” and “IL1_6h”. In TNF-alpha stimulated inflammation, HUVEC were left untreated or stimulated for 5 h with TNF-alpha, and both were repeated for 4 times, thus constructing another 4 sets of data, namely “TNF1”, “TNF2”, “TNF3” and “TNF4”. A total of 8 sets of genes were used for following analysis.

**Figure 2:**
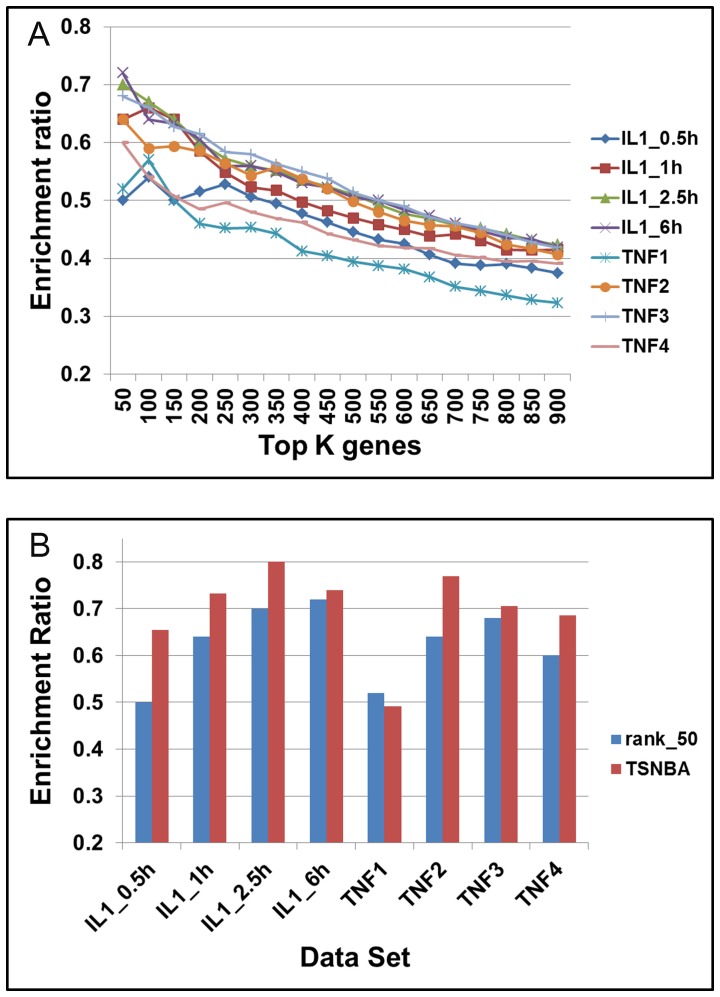
Enrichment analysis of inflammation signature by TSNBA. (A): Enrichment ratio of top K genes for different data sets. IL1_0.5h, IL1_1h, IL1_2.5h, and IL1_6h represent for the perturbation with 0.5, 1.0, 2.5, and 6.0 hours IL-1 stimulation, respectively. TNF1-4 represent for the perturbation with 5 hours TNF stimulation. (B) Comparison of ranking algorithm with TSNBA, rank_50 represents for top 50 ranking genes.

The ranked gene list was first obtained for each data set. The enrichment ratio and p-value were calculated by comparing top **K** ranking genes to the benchmark Entrez inflammation gene set. As shown in Figure 2A, the enrichment ratio (p-value<0.0001) decreased with the increase of **K**, indicating higher probability of finding inflammation gene in the higher ranked genes. The highest enriched ratio was 0.72 in top 50 ranking genes from IL1_6h data set.

As shown in Figure 2A, there were some fluctuations when the number of selected genes ranged from 50 to 250. Taking “TNF1” data set for an example, enrichment ratio of top 100 ranking genes was higher than that of top 50, and enrichment ratio of top 300 was higher than that of top 250. These fluctuations were largely due to the small size of selected genes. Moreover, top 300 ranking genes got better pathology enrichment (which will be detailed in discussion). Given these results, **K**, the number of top ranking genes, was set to be 300. These genes were further used to construct co-expression networks. When the correlation threshold ranged from 0.6 to 0.84, little change in enrichment ratio was observed (see details in Figure S1 in File S1). However, when threshold was larger than 0.84, enrichment ratio increased. We then adjusted threshold to constrain the number of genes in co-expression network to about 50, detailed information was shown in Table 1. Except for “TNF1” data set, TSNBA significantly enriched inflammatory signature further from the top 300 ranking genes. Furthermore, we compared enrichment ratio of TSNBA with that of top 50 ranking genes from step 1. TSNBA outperformed ranking algorithm on 7 out of 8 (87.5%) cases for identifying inflammation enriched signature (as shown in Figure 2B).

**Table 1:** Inflammation enrichment by TSNBA.

Data set	Size of signature	Enrichment ratio	Threshold	P-value
**IL1_0.5h**	52	0.65	0.91	0.006131
**IL1_1h**	56	0.73	0.93	0.000115
**IL1_2.5h**	50	0.80	0.92	2.59E-05
**IL1_6h**	50	0.74	0.93	0.001221
**TNF1**	55	0.49	0.94	0.220595
**TNF2**	52	0.77	0.935	5.80E-05
**TNF3**	51	0.71	0.935	0.014408
**TNF4**	54	0.69	0.94	0.000223

### TSNBA predicted new inflammation related signature genes

The number of genes in co-expression network was set to be about 50, but no less than 50. Under such a restricted condition, the highest enrichment ratio was found in “IL1_2.5h” data set with the threshold of 0.92 (Figure 2B). In the constructed co-expression network, 80% (40/50, p-value<0.0001) genes were confirmed by Entrez Gene database to be human inflammatory genes. Hence, the rest 10 unconfirmed genes were predicted to be inflammatory genes. According to evidence collected from literature, 8 out of the 10 genes were reported to be associated with inflammation (see Table 2 with more details in Table S1 in File S1). Therefore, TSNBA was able to accurately predict pathology related genes by integrating gene expression and PPI network.

**Table 2:** Literature evaluation of predicted inflammation genes.

Potential inflammatory gene	Whether inflammation related gene	Pubmed
**BCL3**	√	19270711
**CALCOCO2**	√	23820297
**CD22**	√	22806142
**DLG3**	Not known	
**ERBB3**	√	22157714
**MAGI1**	√	22806142
**POU1F1**	Not known	
**SMURF2**	√	22843012
**SSTR2**	√	15806094
**TANK**	√	16698233

“√” represents for “Yes”.

### TSNBA predicted key inflammatory regulators

Starting from the top 300 ranking genes, CLR was used to infer regulatory relationships. Potential regulators were predicted for each set and a final union set was obtained. A total of 32 transcription factors were predicted as potential regulators of inflammation. Among them, 21 regulators were included in inflammatory signature from Entrez Gene database and all of them were validated by literature to be important regulators of inflammation, e.g. nuclear factor kappa B (NFκB) a known master regulator of inflammation. In the rest 11 predicted regulators, serum response factor (SRF) was reported to regulate type I interferon-signaling in macrophage, thus is suggested as important regulator for regulating innate immunity [54]. Moreover, SRF was proposed to be required in acrolein activation of NFκB [55]. Macrophage (retinoid X receptor alpha) RXRA could upregulate the expression of chemokines, such as CCL6 and CCL9, and control innate inflammatory responses [56]. Therefore, RXRA and SRF may play important role in the regulation of inflammation. In summary, a total of 71.9% (23/32) regulators predicted by TSNBA were validated by literature as inflammatory regulators (see Table 3 and details in Table S2 in File S1).

**Table 3:** Literature evaluation of predicted inflammatory regulator.

Potential Key Regulator	Whether inflammatory regulator	Whether in benchmark Entrez Gene inflammation set	PubMed
**ATF3**	√	√	18794337
**BCL6**	√	√	22465074
**CEBPB**	√	√	22074460
**EGR1**	√	√	11100120
**ESR2**	√	√	20045727
**FOS**	√	√	19995753
**HES1**	√	√	20832754
**JUNB**	√	√	19933155
**JUND**	√	√	19933155
**NFKB1**	√	√	18927578
**NFKB2**	√	√	18927578
**NR2C2**	√	√	16675448
**POU2F1**	√	√	21059098
**RELA**	√	√	12509469
**SMAD2**	√	√	20667820
**SMAD7**	√	√	19352540
**STAT5A**	√	√	15749913
**TP53**	√	√	21779518
**TP73**	√	√	10716451
**VDR**	√	√	17224129
**VTN**	√	√	17982099
**RXRA**	√		20498053
**SRF**	√		23705899; 23893683
**DLX5**	Not Known		
**GTF2I**	Not Known		
**HEY1**	Not Known		
**MSX1**	Not Known		
**NFE2**	Not Known		
**NR5A1**	Not Known		
**PITX1**	Not Known		
**PRRX1**	Not Known		
**RARA**	Not Known		

“√” represents for “Yes”.

In addition to the validation from the literature evidence, bioinformatics approaches were also taken to explore the relationship between predicted TFs and inflammation. Potential TF binding sites within promoter regions of the 32 TFs were assessed via TRANSFAC component of GATHER [57]. The result showed strong evidence that nuclear factor kappa B (NFκB) binding motifs were contained in the proximal promoter regions of all genes (see details in Table S4 in File S1). Given the critical role of NFκB in regulating inflammation, it is very likely that these regulators are all involved in the process of inflammation. On the other hand, TFactS database [58] was used to find target genes for the rest TFs that were not validated by literature. As a result, 5 of the 6 TFs included in TFactS were reported to target inflammatory genes included in benchmark Entrez Gene database (see details in Table S5 in File S1). Furthermore, these TFs were all shown to interact with confirmed inflammatory proteins via PPIs collected from STRING database (Release 9.1) (see details in Table S6 in File S1) [59].

In summary, both literature and bioinformatics analysis suggested that our predicted TFs were closely related to inflammation, thus indicating the applicability of TSNBA for finding key inflammatory regulators.

### Methods comparison

Statistical significance test was performed on the methods comparison part. According to the working flow of our approach, paired t-test was performed to evaluate statistical significance for results of the first and second step, and enrichment ratios of different sets via different methods were used for comparison.

**Figure 3:**
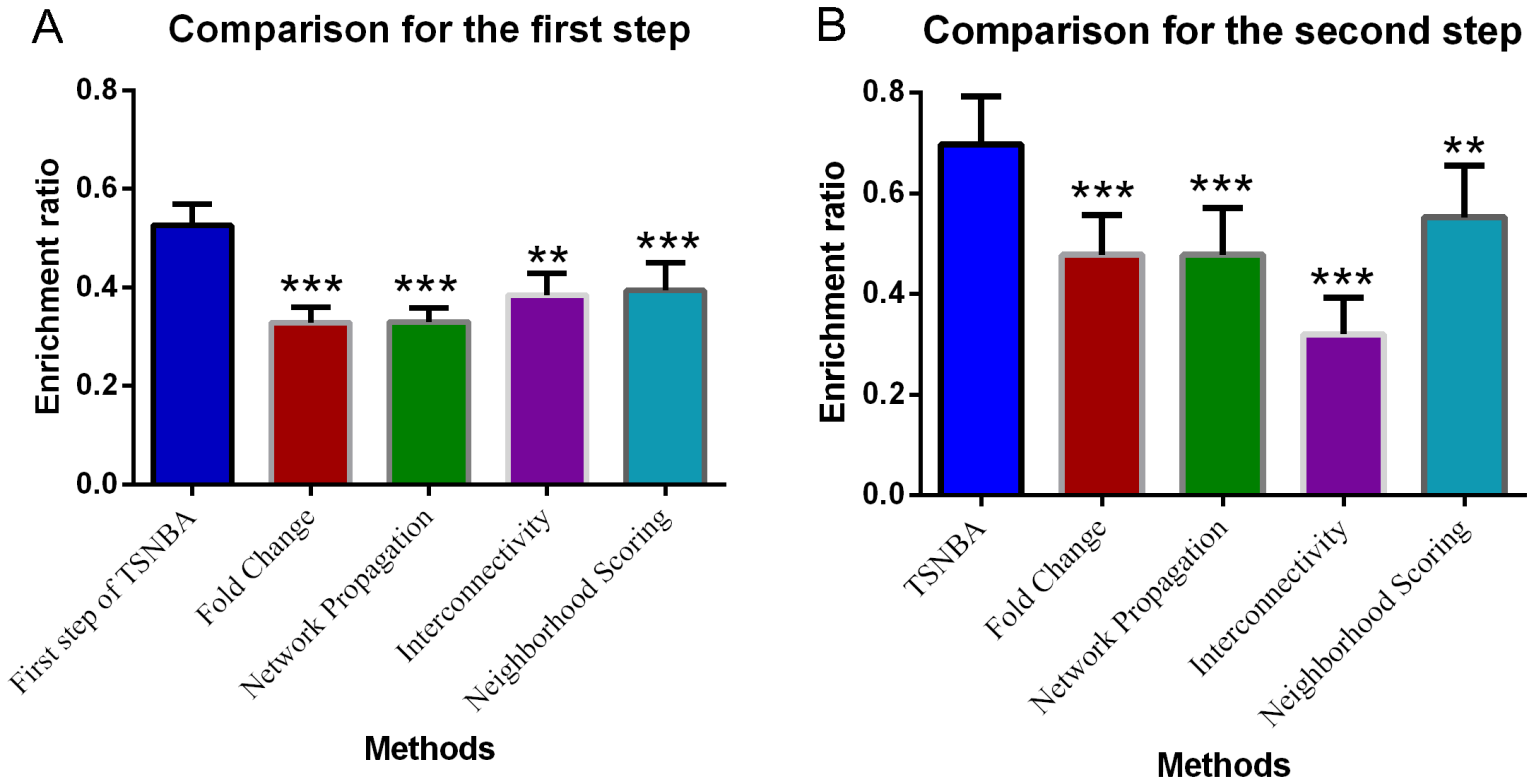
Statistical significance test for methods comparison. Fold change based approach (red), network propagation (green), interconnectivity (purple), and neighborhood scoring (cyan) are compared with first step of TSNBA (A) and full TSNBA (B).

In the first step, top 300 ranking genes were extracted and enrichment ratio was calculated for each data set. As shown in Figure 3A, the first step of TSNBA outperformed another 4 methods (see details in Table S8 in File S1).

**Figure 4:**
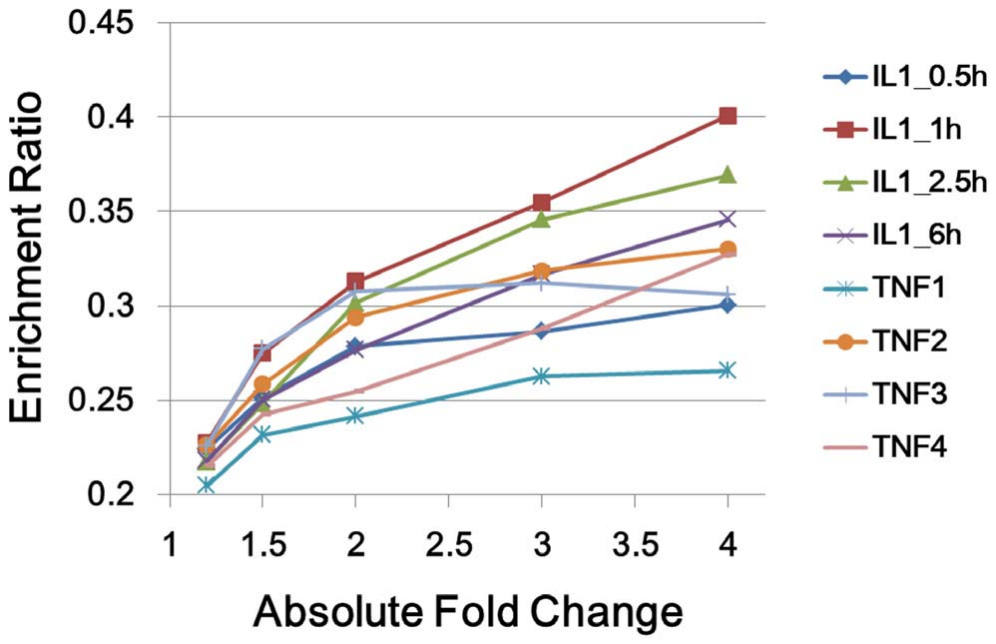
Performance of fold change based approach. Enrichment ratios are calculated under 5 absolute fold change cutoffs, namely 1.2, 1.5, 2.0, 3.0 and 4.0. Different colors represent for different data sets.

**Figure 5:**
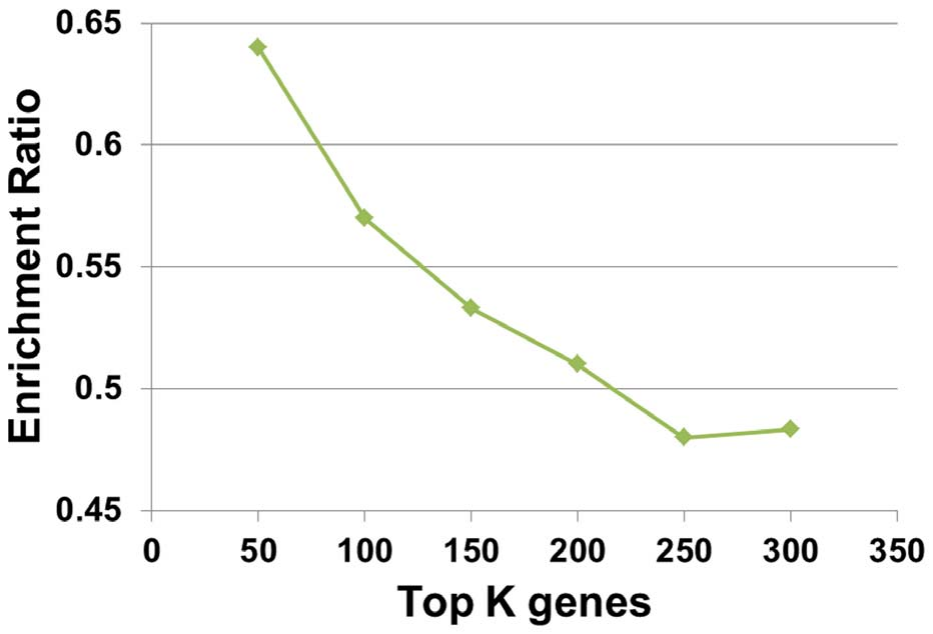
Performance of network degree based approach. Enrichment ratios are calculated for top K genes. The size of genes, K, is set to be 50, 100, 150, 200, 250 and 300.

**Figure 6:**
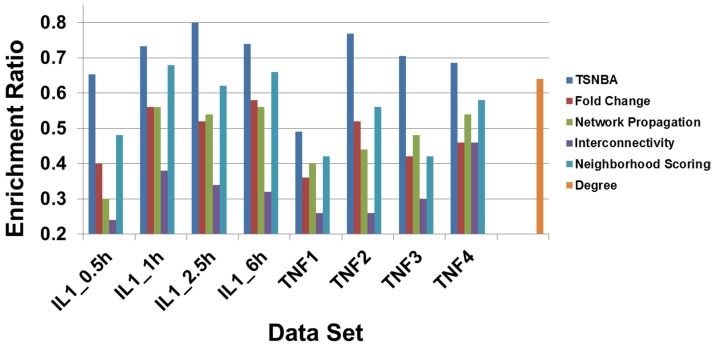
Methods comparison. TSNBA (blue), fold change based approach (red), interconnectivity (purple), neighborhood scoring (cyan) and network propagation (green) are tested on different data sets. To note, the enrichment ratio for degree based approach only depend on the selected network.

In order to perform statistical significance test for the second step of TSNBA, molecular signatures needed to be identified by given methods. When molecular signature was identified by fold change based approach, enrichment ratios were calculated for different data sets under different fold change cutoff. As shown in Figure 4, enrichment ratios increased with increasing cutoff, which indicated that genes with large fold change were more likely to be included in the pathological process. Given this, top 50 ranking genes with highest absolute fold change was set to be molecular signature. As for degree based approach, enrichment ratios for different degree cutoffs were studied. The highest enrichment ratio was obtained under top 50 genes (as shown in Figure 5). Therefore, these 50 genes were identified as the molecular signature. Given results from degree based and fold change based approach, absolute fold change was set to be 4 to identify differentially expressed genes. For the convenience of comparison, the size of signature was set to be 50 for 3 candidate gene prioritization methods. Moreover, diffusion parameter α was investigated (see Table S7 in File S1), and finally set to be 0.1 for best performance. As shown in Figure 3B and Figure 6, TSNBA outperformed fold change based approach, interconnectivity, neighborhood scoring and network propagation in all data sets (see details in Table S9 in File S1). Besides, our approach also outperformed degree based approach in 7 data sets (except for “TNF1” data set).

## Discussion

Proteins play a central role in activities of living cells and they are interconnected in PPI network. Systematic PPI network exploration could lead to a better understanding of protein function and biological processes [60]. Combining PPI network with gene expression profiles provides two advantages. First, microarray gene expression enables genome wide screening of all genes at once and thus guarantees a comprehensive coverage. Second, gene expression data captures perturbation responsive information and perturbation related PPIs are more likely to be activated. Our ranking algorithm takes both aspects into consideration and could find more relevant gene/protein sets. It has long been known that genes involved in the same process often share similar expression patterns. It is thus the motivation of the second step to further reveal the underlying biological process by constructing co-expression network. Co-expression network was used as an integrative filter to find functionally related signature genes. In addition, CLR provided more detailed regulatory information beyond co-expression and identified key regulators of the pathological process. In summary, TSNBA utilized PPI network, co-expression network and regulatory network to progressively find pathology relevant signature genes and regulators.

TSNBA is able to find perturbation responsive molecular signatures. Due to the differences of experimental settings and biological samples, signatures may be different even for the same perturbation. Taking TNF-alpha case for an example, we identified 4 different molecular signatures. Wikipathway enrichment analysis was carried out for each signature by WebGestalt [61], [62]. “hsapiens_entrezgene_protein-coding” was set as the reference set and hypergeometric p-value was calculated. As a result, TNF-alpha signaling pathway was significantly enriched for each signature with adjusted p-value less than 0.01 (see details in Table S3 in File S1). Similarly, IL-1 signaling pathway was also significantly enriched for signatures derived from IL-1 stimulated data sets with adjusted p-value less than 0.05 (see details in Table S3 in File S1). These results provided a good support for the ability of TSNBA in finding perturbation responsive signatures. Moreover, many genes were shared by signatures of the same stimulation. Nine genes (NFKBIA, CALCOO2, PLAU, TNFAIP3, SQSTM1, EGFR, BCL3, BIRC3, BMP2) were shared by 4 TNF-alpha signatures and 13 genes (NFKBIA, JUNB, FASLG, NFKB2, PLAU, CCL5, ATF3, BCL6, CTNNB1, PRTN3, BCL2A1, BMP2, ERBB3) by 4 IL-1 signatures.

As shown in Figure 5, it should be noted that enrichment ratio generally decreased with the size of top ranking genes, which suggested that degree information was associated with enrichment. However, ranking algorithm, which guaranteed the improvement in enrichment for our approach, inadvertently captured degree information (see the method). Is improvement in enrichment largely attributed to degree information and do these ranking lists worth further study? Therefore, in order to further evaluate our algorithm's ability in finding perturbation responsive signature, wiki pathway enrichment analysis was performed for top 300 ranking genes by degree based approach and our approach, respectively. As shown in Table 4, our algorithm outperformed degree based approach in all data sets by ranking perturbation responsive pathway higher. Moreover, in “TNF1” and “TNF2” data sets, our approach even ranked the “TNF alpha Signaling Pathway” the number one, which was a strong indication of the reliability of our approach. Besides, top 300 highest degree genes were compared with molecular signatures identified by TSNBA, less than 33% of genes were overlapped, suggesting that network degree was not the major contributor of the high enrichment of TSNBA.

**Table 4:** Comparison of TSNBA and Degree based approach for top 300 ranking genes.

Data set	Pathway Name	Rank (Selected data set vs Degree)
**IL1_0.5h**	IL-1 signaling pathway	27 vs 43
**IL1_1h**	IL-1 signaling pathway	9 vs 43
**IL1_2.5h**	IL-1 signaling pathway	34 vs 43
**IL1_6h**	IL-1 signaling pathway	18 vs 43
**TNF1**	TNF alpha Signaling Pathway	1 vs 18
**TNF2**	TNF alpha Signaling Pathway	1 vs 18
**TNF3**	TNF alpha Signaling Pathway	6 vs 18
**TNF4**	TNF alpha Signaling Pathway	14 vs 18

An inspection of the results presented in Figure 2B and Figure 6 showed that degree and fold change methods showed the results on TNF2, TNF3 and TNF4 to be similar with TNF1 being particularly less. TSNBA also showed the lowest enrichment result on TNF1 data set. Our approach took the fold change into consideration in the first step, and fold change was an important factor to rank genes. Besides, as shown in Figure 4, fold change based approach also performed worst in TNF1 data set, which indicated that the worst performance in “TNF1” data set was likely due to the data itself.

In addition, we took union set of signatures of different conditions by fold change based approach, interconnectivity, neighborhood scoring, network propagation, and TSNBA. The size of these sets was 242, 247, 233, 265 and 192 (see details in Table S10 in File S1). This indicated that more genes were overlapped in signatures determined by TSNBA, and our method was more likely to find reproducible signatures. TNFRSF9 and TRAF1 were shared by all sets, and 19 genes (PLAU, ALOX12, ATF3, POU1F1, FOS, CSF1, CCL8, JUNB, TNFRSF11B, ICAM1, SELE, VCAM1, IL8, NFKBIA, RND1, TNFAIP3, BCL2A1, CSF2, and BIRC3) were shared by 4 sets except for the sets determined by interconnectivity, while 4 genes (OCM2, CLEC2D, MATN3, and IFIT3) were shared by other 4 sets but not the set of our approach. A recent report had pointed out the regulatory role of MATN3 in inducing the IL-1Ra and raised the possibility of recombinant human MATN3 protein in anti-inflammatory therapy [63]. Therefore, there were still some important inflammation related genes that may be missed by our approach, and common signature of several methods was worth of follow up investigation.

Gene expression profile has been used widely to represent indirectly the protein activity. It has its limitations in quantifying actual protein abundance and incapable of reflecting many ‘switches’ in PPI behavior, such as ligand binding and posttranslational modification [64]. Therefore, other data types, such as protein and microRNA expression profiles should be integrated to further reveal these missing actions. At the same time, the network should be updated. Ever-increasing amount of PPIs shall continuously be incorporated into the network. In addition, many other interaction types, such as DNA-protein interaction, transcription factor-target interaction and microRNA-target interaction, shall also be included. It could be envisioned that a comprehensive network with biologically relevant profiles will lead us to more accurate disease molecular signature finding.

## Conclusion

In this study, TSNBA was proposed to identify the molecular signature and key pathological regulators. In the case study of IL-1 and TNF-alpha stimulated inflammation, TSNBA identified inflammatory signature with high enrichment of pathology related genes and outperformed 5 methods in prioritizing candidates, including fold change based approach, degree based approach, interconnectivity, neighborhood score and network propagation. In conclusion, we developed an efficient strategy for disease molecular signature finding and key pathological regulator identification.

## Supporting Information

File S1 Including the following: (1) Enrichment ratios under different thresholds in the second step (Figure S1); (2) literature evaluation for 10 predicted inflammatory genes (Table S1); (3) literature evaluation for 32 predicted regulators of inflammation (Table S2); (4) wikipathway enrichment analysis of molecular signatures via TSNBA (Table S3); (5) TF binding sites analysis of 32 predicted regulators of inflammation (Table S4); (6) transcription factor-inflammatory gene relationships from TFactS for 5 transcription factors, namely DLX5, MSX1, NR5A1, PRRX1, and RXRA (Table S5); (7) protein-protein interactions collected from STRING database for 9 transcription factors that are confirmed by literature to be inflammatory regulators (Table S6); (8) investigation for diffusion parameter α of network propagation (Table S7); (9) methods comparison for the first step of TSNBA (Table S8); (10) methods comparison for TSNBA (Table S9); (11) union sets of signatures from different methods.
(XLSX)
